# Supplementary material for: Time-series transcriptome analysis identified differentially expressed genes in broiler chicken infected with mixed Eimeria species
Source: Front Genet. 2022 Aug 8;13:886781. doi: 10.3389/fgene.2022.886781 (PMC9393255; doi:10.3389/fgene.2022.886781)
Supplement: Supplementary file 2 [file DataSheet1.ZIP › 4dpi_KEGG.Gsea.1625100298584/KEGG_DRUG_METABOLISM_CYTOCHROME_P450.html]

Details for gene set KEGG\_DRUG\_METABOLISM\_CYTOCHROME\_P450[GSEA]

|  || Dataset | TMM\_4dpi\_gct\_format\_4dpi\_gct\_format.Class\_4dpi.cls #PC\_versus\_NC.Class\_4dpi.cls #PC\_versus\_NC\_repos |
| Phenotype | Class\_4dpi.cls#PC\_versus\_NC\_repos |
| Upregulated in class | 0 |
| GeneSet | KEGG\_DRUG\_METABOLISM\_CYTOCHROME\_P450 |
| Enrichment Score (ES) | -0.49014163 |
| Normalized Enrichment Score (NES) | -1.5545136 |
| Nominal p-value | 0.022789426 |
| FDR q-value | 0.19590749 |
| FWER p-Value | 0.8406 |
Table: GSEA Results Summary

  

Fig 1: Enrichment plot: KEGG\_DRUG\_METABOLISM\_CYTOCHROME\_P450      
 Profile of the Running ES Score & Positions of GeneSet Members on the Rank Ordered List

  

| SYMBOL | TITLE | RANK IN GENE LIST | RANK METRIC SCORE | RUNNING ES | CORE ENRICHMENT || 1 | CYP2D6 | na | 49 | 2.025 | 0.1127 | No |
| 2 | ALDH1A3 | na | 581 | 0.895 | 0.1200 | No |
| 3 | GSTT1 | na | 1493 | 0.521 | 0.0741 | No |
| 4 | FMO3 | na | 1581 | 0.500 | 0.0957 | No |
| 5 | GSTM2 | na | 2462 | 0.353 | 0.0426 | No |
| 6 | MAOB | na | 3877 | 0.180 | -0.0650 | No |
| 7 | MAOA | na | 5750 | 0.013 | -0.2204 | No |
| 8 | MGST2 | na | 5918 | -0.001 | -0.2343 | No |
| 9 | GSTZ1 | na | 7777 | -0.159 | -0.3802 | No |
| 10 | CYP3A5 | na | 8491 | -0.228 | -0.4265 | No |
| 11 | GSTK1 | na | 9125 | -0.299 | -0.4621 | No |
| 12 | GSTO2 | na | 9462 | -0.343 | -0.4704 | Yes |
| 13 | CYP3A4 | na | 9602 | -0.362 | -0.4611 | Yes |
| 14 | ADH6 | na | 9642 | -0.367 | -0.4431 | Yes |
| 15 | UGT2A1 | na | 10045 | -0.421 | -0.4524 | Yes |
| 16 | MGST3 | na | 10072 | -0.424 | -0.4301 | Yes |
| 17 | ALDH3B2 | na | 10162 | -0.439 | -0.4122 | Yes |
| 18 | GSTA2 | na | 10745 | -0.548 | -0.4291 | Yes |
| 19 | MGST1 | na | 11272 | -0.692 | -0.4331 | Yes |
| 20 | GSTA3 | na | 11653 | -0.916 | -0.4120 | Yes |
| 21 | AOX1 | na | 11728 | -0.999 | -0.3606 | Yes |
| 22 | ADH4 | na | 11768 | -1.035 | -0.3041 | Yes |
| 23 | ADH1C | na | 11810 | -1.129 | -0.2424 | Yes |
| 24 | CYP2C18 | na | 11900 | -1.289 | -0.1755 | Yes |
| 25 | UGT1A1 | na | 11907 | -1.313 | -0.1002 | Yes |
| 26 | GSTA4 | na | 11979 | -1.885 | 0.0026 | Yes |
Table: GSEA details [plain text format]

  

Fig 2: KEGG\_DRUG\_METABOLISM\_CYTOCHROME\_P450      
 Blue-Pink O' Gram in the Space of the Analyzed GeneSet

  

Fig 3: KEGG\_DRUG\_METABOLISM\_CYTOCHROME\_P450: Random ES distribution      
 Gene set null distribution of ES for **KEGG\_DRUG\_METABOLISM\_CYTOCHROME\_P450**
